# Neurocysticercosis as an important differential of seizures in pregnancy: two case reports

**DOI:** 10.1186/1752-1947-5-206

**Published:** 2011-05-26

**Authors:** Savita R Singhal, Smiti Nanda, Suresh K Singhal

**Affiliations:** 1 Department of Obstetrics and Gynecology, Pandit Bhagwat Dayal Sharma, Post Graduate Institute of Medical Sciences, Rohtak (124001), Haryana, India; 2 Department of Anesthesiology, Pandit Bhagwat Dayal Sharma, Post Graduate Institute of Medical Sciences, Rohtak (124001), Haryana, India

## Abstract

**Introduction:**

Seizures in pregnancy usually result from eclampsia, epilepsy or central nervous system disorders. Neurocysticercosis is a rare, but an important, cause of first-time convulsions in pregnancy.

**Case presentations:**

We report the cases of two Indian women, aged 20 and 24 years old respectively, with neurocysticercosis presenting in the second trimester of pregnancy with convulsions. Both had marginally raised blood pressure with 1+ urine albumin and neither had a past history of convulsions. The neurocysticercosis was diagnosed on magnetic resonance imaging of the head, which showed spherical ring-enhancing lesions in the brain. In one woman, pregnancy was terminated due to spina bifida in the fetus and she was discharged on albendazole and phenytoin. The second woman was put on carbamazepine: she had an emergency Cesarean section at term for fetal distress and delivered a healthy baby. Her postnatal period was uneventful.

**Conclusion:**

Neurocysticercosis should be considered in pregnant women presenting with seizures which cannot be explained by eclampsia, especially in early pregnancy.

## Introduction

Seizures in pregnancy usually result from eclampsia, epilepsy or central nervous system disorders. Neurocysticercosis, although rare, is an important cause of first-time convulsions in pregnancy. Del Brutto has proposed certain definitive and probable criteria for the diagnosis of neurocysticercosis: histology; imaging; epidemiology; serology; clinical symptoms; and follow-up scans [1]. Magnetic response imaging (MRI) is superior to a computed tomography (CT) scan in diagnosis and follow-up studies [2]. The signs and symptoms range from a single seizure to coma and death. It can be treated with minimal interruption to the course of the pregnancy and medical treatment is effective in most cases although surgery may be indicated for a few women [3]. We report two cases of neurocysticercosis in women who presented with convulsions in the second trimester of their pregnancy.

## Case presentations

### Case 1

A 20-year-old Indian woman who was an unbooked primigravida presented as an emergency at 27 weeks of gestation with generalised tonic clonic seizures over a six hour period. She had been unconsciousness for one hour. She was referred by a general practitioner with a diagnosis of eclampsia. There was no past history of seizures and she was not on any medication. On examination, she was in a grade III coma and her pulse and blood pressure were 92 beats/min and 130/90 mmHg, respectively. Her urine albumin was +1 and the liver function, renal function and fundus examinations were normal. An ultrasound showed a live 26-week-size fetus with spina bifida. An emergency MRI of our patient's head showed a 4-5 mm spherical ring-enhancing lesion in the frontal region of her brain. A diagnosis of neurocysticercosis was made on the basis of the MRI finding, symptoms and living in an endemic area. She was put on intravenous phenytoin and the pregnancy was terminated in view of the fetus' spina bifida with misoprostol. She aborted after 16 hours and regained consciousness after 24 hours: she was discharged on albendazole and phenytoin.

### Case 2

**Figure 1:**
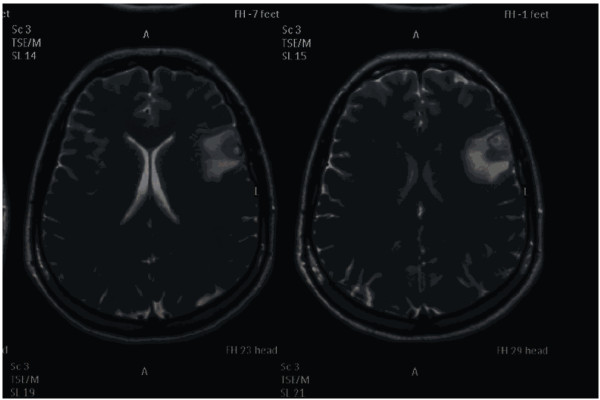
**MRI of the brain of the patient in case 2 showing a single cystic enhanced lesion**.

A 24-year-old Indian woman who was a booked primigravida presented at 24 weeks of gestation with a history of one generalized tonic clonic seizure 30 minutes before. She had no past history of convulsion. On examination she was conscious, alert and had a blood pressure of 140/100 mmHg. There was +1 albuminuria. Fundus, renal and liver function tests were normal. She became normotensive after 24 hours. An MRI of her head reported a single cystic enhanced lesion in the parietal lobe of the brain (Figure 1). A diagnosis of neurocysticercosis was made on the basis of the MRI, convulsion and living in an endemic area. She was put on carbamazepine and discharged from the hospital after two days. She was followed-up regularly throughout the pregnancy. The remainder of her antenatal period was uneventful but, at term, she had an emergency Cesarean section due to fetal distress. Her postnatal period was uneventful and she was discharged in good condition with a healthy baby.

## Discussion

Convulsions during pregnancy reported to an obstetrician are mainly due to eclampsia. A complete and continuous evaluation is crucial to differentiate eclampsia from other disorders. When atypical features are present, or clinical status worsens, neuroimaging is important [4]. In both these reported cases there was marginally raised blood pressure; +1 albuminuria; no past history of any seizures; and both women presented in the second trimester. Marginally raised blood pressure and +1 urine albumin may be present in acute stages of convulsions. In both women, the MRI was suggestive of neurocysticercosis.

The first case was misdiagnosed as eclampsia at periphery. There was a small possibility of eclampsia as her blood pressure was only 130/90 mmHg and only +1 urine albumin. Bearing this in the mind, an emergency MRI of the head was done which detected the spherical ring-enhancing lesion. The diagnosis of neurocysticercosis was made on the basis of MRI findings, symptoms and living in an endemic area. She presented in a very bad state with a grade III coma as she had multiple seizures over a period of six hours. The second woman was in quite good condition in spite of having suffered a seizure. Thus, the presentation may be variable from a single episode to multiple seizures.

Neurocysticercosis is usually misdiagnosed as eclampsia and can be differentiated by imaging studies as in these two cases. MRI is superior to a CT scan in such cases [2]. During pregnancy, treatment of neurocysticercosis consists of anti-convulsant therapy. Anti-helminthic drugs should be delayed until post-partum [5].

## Conclusion

Neurocysticercosis should be considered as a possible diagnosis in pregnant women presenting with seizures which cannot be explained by eclampsia, especially in early pregnancy.

## Consent

Written informed consent was obtained from both patients for publication of these case reports and any accompanying images. A copy of the written consent is available for review by the Editor-in-Chief of this journal.

## Competing interests

The authors declare that they have no competing interests.

## Authors' contributions

Both the patients were admitted under the supervision of SN. SRS did the Cesarean section for one patient and terminated the pregnancy in the other. SKS gave anesthesia to the patient and helped to collect the literature for this report. SRS and SN wrote the article. All the authors have read and approved the final manuscript.
